# Dual opposite actions of sodium bicarbonate in treatment of acute organophosphate poisoning

**DOI:** 10.17179/excli2019-1687

**Published:** 2019-08-23

**Authors:** Seyed Vahid Shetab-Boushehri

**Affiliations:** 1 Department of Toxicology and Pharmacology, School of Pharmacy, International Campus, and Razi Drug Research Center, Iran University of Medical Sciences, Tehran, I.R. Iran

## ⁯

***Dear Editor,***

Organophosphate pesticides (OPs), triesters of phosphoric or thiophosphoric acids, have been well known to inhibit both red cell (true or acetyl) and plasma (pseudo or butyryl) cholinesterases (ChEs) and induce poisoning in human (Shetab-Boushehri 2018[7]; Shetab-Boushehri et al., 2012[8]). Recently, some researchers have shown that sodium bicarbonate increases survival of OP-poisoned individuals (Balali-Mood et al., 2005[2]). In a 'Letter to Editor', some authors commented on and criticized the application of sodium bicarbonate in this article (Hittarage et al., 2007[3]). Other researchers performed a systematic review through the Cochrane Database of Systematic Reviews which indicated that there was insufficient evidence to recommend the routine use of blood alkalization in acute OP pesticide poisoning (Roberts and Buckley, 2005[6]). The authors of the criticized paper replied to them in another 'Letter to Editor' that more patients should be studied to obtain more conclusive results, as the sample size of their study was small (Balali-Mood et al., 2007[1]). The IPCS working group on antidotes for organophosphorus pesticide poisoning of The World Health Organization has also highlighted the potential efficacy of sodium bicarbonate as an antidote for treatment of OP poisonings (Johnson et al., 2000[4]). Theoretically, sodium bicarbonate may exert its beneficial effect in treatment of OP poisoning by two mechanisms: the first is deactivation (dealkylation) of OPs by alkalinization of blood through a direct action and the second is reactivation of ChEs by breaking OP-ChE bond (Mohammadi et al., 2017[5]). The first pathway actually occurs because OP triesters are very sensitive to basic pH and are converted to di-substituted counterparts which are less reactive toward OPs. The second one does not actually occur as alkalinization of blood dealkylates OP moiety of OP-ChE complex which results in aging of ChEs and deterioration of poisoning (Shetab-Boushehri et al., 2012[8]; Mohammadi et al., 2017[5]). Moreover, the authors of the criticized paper showed no reactivation of inhibited ChEs by sodium bicarbonate (Balali-Mood et al., 2005[2]). As a matter of fact, sodium bicarbonate has dual opposite actions in treatment of OP poisoning. It seems that the time of sodium bicarbonate administration in OP poisoning plays an important role in the outcome of treatment. To reaching highest effect, sodium bicarbonate seems to be administered in early stages of OP poisoning and the pH of the blood should be brought to the highest allowable limit. After saturation of all available free ChEs active sites by OPs, alkalinization of blood by sodium bicarbonate seems to produce a reverse effect namely, increasing the risk of ChE aging (Shetab-Boushehri et al., 2012[8]). 

## Conflict of interest

The author declares no conflict of interest.
